# Potential Consequences of the Use of Adipose-Derived Stem Cells in the Treatment of Hepatocellular Carcinoma

**DOI:** 10.3390/ijms25147806

**Published:** 2024-07-17

**Authors:** Aleksandra Gładyś, Adam Mazurski, Piotr Czekaj

**Affiliations:** 1 Department of Cytophysiology, Chair of Histology and Embryology, Faculty of Medical Sciences in Katowice, Medical University of Silesia in Katowice, 40-752 Katowice, Poland; d201163@365.sum.edu.pl; 2 Students Scientific Society, Chair of Histology and Embryology, Faculty of Medical Sciences in Katowice, Medical University of Silesia in Katowice, 40-752 Katowice, Poland; s74807@365.sum.edu.pl

**Keywords:** hepatocellular carcinoma, adipose-derived stem cells, conditioned medium, anti-cancer therapy, cell-based therapy

## Abstract

Hepatocellular carcinoma (HCC) ranks as the most prevalent of primary liver cancers and stands as the third leading cause of cancer-related deaths. Early-stage HCC can be effectively managed with available treatment modalities ranging from invasive techniques, such as liver resection and thermoablation, to systemic therapies primarily employing tyrosine kinase inhibitors. Unfortunately, these interventions take a significant toll on the body, either through physical trauma or the adverse effects of pharmacotherapy. Consequently, there is an understandable drive to develop novel HCC therapies. Adipose-derived stem cells (ADSCs) are a promising therapeutic tool. Their facile extraction process, coupled with the distinctive immunomodulatory capabilities of their secretome, make them an intriguing subject for investigation in both oncology and regenerative medicine. The factors they produce are both enzymes affecting the extracellular matrix (specifically, metalloproteinases and their inhibitors) as well as cytokines and growth factors affecting cell proliferation and invasiveness. So far, the interactions observed with various cancer cell types have not led to clear conclusions. The evidence shows both inhibitory and stimulatory effects on tumor growth. Notably, these effects appear to be dependent on the tumor type, prompting speculation regarding their potential inhibitory impact on HCC. This review briefly synthesizes findings from preclinical and clinical studies examining the effects of ADSCs on cancers, with a specific focus on HCC, and emphasizes the need for further research.

## 1. Introduction

Liver cancers are a major challenge for public health, as they rank third in mortality and sixth in incidence of all malignant neoplasms. Both incidence and mortality are expected to rise by over 50% by the year 2040. Among liver cancers, the most common subtype is hepatocellular carcinoma (HCC), which accounts for 80% of cases [1]. HCC is usually preceded by liver damage caused by alcohol consumption, non-alcoholic fatty liver disease (NAFLD), non-alcoholic steatohepatitis, and hepatitis B or C [2].

The treatment of hepatocellular carcinoma depends on the stage of the disease at the time of diagnosis and preserved liver function. For early and intermediate HCC, treatment options usually include liver resection, orthotopic liver transplantation, or the thermal ablation of the tumor. Advanced HCC requires systemic treatment. HCC is generally resistant to chemotherapy, therefore the first-line treatment in advanced HCC uses tyrosine kinase inhibitors, such as sorafenib [3]. Immunotherapy is emerging as a new potential alternative in the treatment of HCC, mainly in the United States, where it has been accepted as the first-line therapy [4,5]. Radiotherapy is not widely used in HCC, but it may be an alternative in patients who cannot be treated with other therapeutic methods [3,6].

Due to the poor prognosis in advanced hepatocellular carcinoma, new treatment opportunities are being investigated. One such proposal may be stem cell therapy. Cell therapy in cancers is the subject of in vitro and in vivo studies [7] as well as clinical trials [8]. Various types of stem cells are widely used in regenerative medicine, especially in wound healing and diseases involving increased inflammation [9]. Malignant tumors are considered physiologically similar to chronic, non-healing wounds, characterized by the increased production of inflammatory substances, hypoxia, and intense oxidative stress. Moreover, they can also cause actual wounds, leading to ulceration or necrosis, and it is thought that the regenerative potential of stem cells may be useful in treating these diseases and the damage they cause to the body [7]. In patients with non-malignant conditions, such as COVID-19, some mesenchymal stromal/stem cells (MSCs) have been shown to reduce the concentrations of pro-inflammatory cytokines and acute phase proteins such as interleukin 6 and 8 (IL-6 and IL-8), tumor necrosis factor α (TNF-α), C-reactive protein (CRP), and D-dimers, while imaging examinations have shown accelerated regeneration of parenchymal organs [10,11,12]. 

## 2. Stem Cells as a Novel Cancer Treatment

Significant scientific evidence suggests the usefulness of various stem cells in the treatment of cancer. Some stem cell therapies, particularly hematopoietic stem cell (HSC) treatment for leukemias and other blood malignancies, are already established as standard practices. HSC transplantation preceded by high-dose chemotherapy is often the gold standard in the treatment of these diseases [13]. Besides hematological malignancies, HSC has demonstrated certain efficacy in the treatment of breast, lung, ovarian, and renal cancers [13,14]. For example, in advanced breast cancer, high-dose chemotherapy followed by HSC provided longer survival in the group of patients with higher-risk cancer (≥10 lymph nodes involved) compared to standard-dose chemotherapy without HSCs; however, this procedure did not provide longer survival in the overall group of patients [8].

Studies on MSCs have shown that these cells exhibit tropism to cancer cells and a propensity to colonize tumors [15], and preclinical studies have demonstrated their anti-proliferative and pro-apoptotic effects on cancer cells. The exact mechanisms responsible for these effects have yet to be elucidated, and unfortunately, only a few clinical studies are being conducted on the potential use of MSCs in the treatment of solid tumors. One phase I study (NCT02530047) concerning ovarian cancer involved genetically modified MSCs secreting interferon β (IFNβ). The ongoing phase I/II trial NCT02068794 also focuses on ovarian cancer but includes oncolytic measles virus (MV-NIS)-infected adipose-derived stem cells (ADSCs). MV-NIS is a modified strain of the measles virus that has oncolytic effects and stimulates the T-cell response against tumor cells [16]. Another ongoing trial is investigating the effect of umbilical cord-derived MSCs modified to express the tumor necrosis factor-related apoptosis-inducing ligand (TRAIL) on lung adenocarcinoma (NCT03298763) [17]. The one completed phase 1 trial (NCT01844661) examined the effects of autologous bone marrow-derived MSCs (BM-MSCs) infected with the oncolytic adenovirus ICOVIR5 on various metastatic and refractory tumors in children and adults. These cells were shown to allow large amounts of the virus to be safely stored in the body, and some patients demonstrated disease stabilization in response to the treatment with BM-MSCs [18].

## 3. ADSC Characteristics

ADSCs are a population of adult multipotent stem cells of mesodermal origin [19], which can be easily extracted from adipose tissue in large amounts [20]. ADSCs are spindle-shaped [21] and express markers typical for mesenchymal stem cells, such as CD44, CD73, CD90, and CD105 [22,23]. Unlike other mesenchymal stem cells, ADSCs at early passages express CD34, a surface marker whose expression decreases in further cultures [20,22]. ADSCs are capable of self-renewal [19] and differentiation towards cells representing all three germ layers [22]. ADSCs are obtained mainly by liposuction (lipoaspiration) [24,25] but also from tissues excised during plastic surgeries, such as abdominoplasty or dermolipectomy [21,23]. The excision of intact tissue provides a higher yield of ADSCs [21] or other adipose tissue cells [26]. ADSCs are usually extracted from a tissue sample via enzymatic digestion with collagenases [21,23], which can also be supported by mechanical sorting [21].

Recent studies have developed a concept in which ADSCs may differ within their subpopulations based on their anatomical origin. For instance, ADSCs from abdominal and gluteofemoral adipose tissue were found to show significant differences in RNA expression, which may play a role in cell metabolism [27]. RNA expression differs not only between ADSCs of different origins but also in other cell populations, such as immune cells derived from visceral or subcutaneous adipose tissue [28]. Similar results for ADSC subpopulations were obtained by Nahmgoong et al., who investigated the differentiation potential, metabolic features, and interactions with the microenvironment of ADSCs derived from visceral epididymal adipose tissue and subcutaneous inguinal adipose tissue [29]. A study on ADSC spheroids distinguished three subpopulations of subcutaneous fat: superficial, deep, and the superficial retinacula cutis [30].

The regenerative properties of various ADSC subtypes make them a recognized field of research in trauma regeneration. Particularly great potential lies in the use of autologous grafts of subcutaneous ADSCs or their exosomes in promoting scar-free wound healing and fractured bone fusion, with a strong focus on the treatment of complications such as aseptic non-unions [31,32]. Tendinopathies and tendon injuries are also areas where ADSCs appear to show significant effectiveness. ADSCs derived from the buttocks, ADSCs from visceral fat, periumbilical abdominal ADSCs, and ADSCs from thigh adipose tissue have all been used in clinical trials for these indications [33]. The clinical application of ADSCs is not limited to the treatment of injuries. In the setting of chronic diseases, they have shown efficacy in the management of many complications of diabetes, including skin ulcers and ophthalmic disorders involving subcutaneous ADSCs and eyelid ADSCs, respectively [34,35].

Furthermore, a large body of evidence, including a recent randomized, double-blind study by Tak et al., indicates the efficacy of ADSC-derived constituent extracts in the treatment of androgenetic alopecia [36]. The results from preclinical studies suggest that ADSCs modified for overexpressing miR-188-3p may be helpful in the treatment of Parkinson’s disease [37]. ADSCs grafts, along with autologous fat grafting and visceral stromal fraction administration, appear to be efficient in systemic sclerosis treatment, suggesting the broader applicability of ADSCs in rheumatology [38]. Abdominal ADSC-derived nanovesicles have been shown to affect nucleus pulposus cells in patients with intervertebral disc degeneration, reducing the severity of inflammation and painful symptoms [39].

The anti-inflammatory properties of ADSCs make them an attractive therapeutic agent [40]. It has been postulated that by regulating immune function, mainly by reducing lymphocytic infiltration in transplanted organs, ADSCs may alleviate the course of graft versus host disease [41], which is a serious side effect of organ transplantation. In clinical settings, the autologous transplantation of human ADSCs is immunologically safe and has positive effects on, for example, wound healing [42] and spinal cord injury [43]. Another alternative may be an allogeneic transplant or the use of the ADSC secretome [44]. Due to their multipotent properties, when cultured with small molecules and cell factors, human ADSCs have the ability to differentiate into cells of mesenchymal, ectodermal (neurons and Schwann cells) [45], and endodermal origin, including functional hepatocyte-like cells [46,47]. The effect of differentiation towards hepatocyte-like cells was improved with the use of laminin-containing scaffolds [47]. ADSCs alleviated liver damage in numerous in vivo studies in animal models of liver fibrosis [48,49,50,51], non-alcoholic fatty liver disease (NAFLD) [52], ischemia-reperfusion injury [53], and liver transplantation [54]. The wound-healing properties of ADSCs may significantly increase the regenerative potential of the liver after surgery, ablation, or embolization.

The regulatory and immunomodulatory properties of ADSCs are mostly due to the factors they secrete into the extracellular matrix (ECM). The ADSC secretome consists of three main components: membrane proteins (including major histocompatibility complex antigens), soluble agents in the form of cytokines and growth factors, and nucleic acid fragments [55,56]. These substances are released into the environment immediately or via exosomes and are able to affect inflammation directly by reversing the effects of pro-inflammatory cytokines or indirectly by regulating the activity of specific populations of immune and other cells. 

Notable substances produced by ADSCs and other MSCs include hepatocyte growth factor (HGF), which enhances regeneration, especially of the parenchymal organs, such as the liver and lungs, vascular endothelial growth factor (VEGF), which promotes angiogenesis [55,57,58,59], and transforming growth factor β (TGFβ), which stimulates tissue regeneration and regulates T-cell activity [60,61]. The immunosuppressive components produced by ADSCs include prostaglandin E2 (PGE2) and indoleamine 2,3-dioxygenase (IDO) [62,63,64]. The components identified in the ADSC secretome are the tissue inhibitors of metalloproteinases TIMP1 and TIMP2 [55], which reduce NK cell activity and are involved in immunosuppression of tumorigenic processes [65]. Interestingly, some of these factors, such as HGF, have also been described as promoting tumor growth [66,67]. In turn, one contribution of ADSCs to cell migration is through their impact on the ICAM-1/RANTES pathway [68].

The ADSC secretome contains significant amounts of various types of collagen, including type I, III, VI, and XII collagen chains [69]. Indirectly, ADSCs increase collagen content in the ECM of cartilage tissue by secreting insulin-like growth factor 1 (IGF-1) and TGFβ, which stimulate chondrocytes to produce type II collagen, proteoglycans, and aggrecans [24]. The secretome of ADSCs also contains other components of the ECM that consolidate its structure and are essential for regenerating damage, including fibronectin, vimentin, laminin, and fibrillin [69].

ADSCs produce various substances that affect the ECM, but it seems that the ECM can also simultaneously affect these stem cells: the enzymes present in the matrix and their inhibitors have been shown to influence the direction of MSC differentiation towards adipocytes, osteoblasts, or chondroblasts [70]. In a study of pancreatic cancer in mice, mucins produced by cancer cells induced the proliferation and migration of ADSCs [71]. An additional factor affecting ADSCs is obesity, which influences the cell metabolism of ADSCs in mammary fat [72]. Similar relationships have been observed in epicardial adipose tissue ADSCs in diabetes, and diabetic ADSCs play a role in cardiovascular disease development [73]. The exact nature of the interaction between ADSC subpopulations and the ECM is still not comprehensively understood and promises significant discoveries in the future.

## 4. Advantages and Disadvantages of the Therapeutic Use of ADSCs in Cancer Therapy 

ADSCs possess several advantages over MSCs isolated from other sources. Their superior safety profile, easy and safe extraction, and exceptional immunomodulatory properties distinguish themselves among stem cell populations. ADSCs are much easier to obtain than some other types of MSCs: adipose tissue, collected through liposuction, is a rich source of stem cells, and the procedure itself is much less traumatic than, for example, bone marrow extraction [74]. Some other features of ADSCs should also be considered, such as their outstanding ability to stimulate the proliferation and differentiation of target cells as well as their ability to stimulate senescent MSCs in surrounding tissue [19].

It has been concluded in some studies that ADSCs represent a high safety profile in cancer treatment because they inhibit tumor growth. However, this effect was mainly shown in in vitro or in vivo xenograft models, and some researchers have questioned the safety of ADSC use in cancer patients [75]. The anti-tumorigenic effect of ADSCs was proven in different types of cancer, such as thyroid [76], bladder [77], prostate [78,79], ovarian [80], colon [81], and gastric cancers [82], as well as in hepatocellular carcinoma [83,84,85,86]. Another study found no harmful effects of ADSCs when cultured in vitro with HSC-3 squamous cell carcinoma cells, which was in contrast to the bone marrow-derived MSCs (BM-MSCs) also examined in that study, which seemed to promote the invasion of cancer cells and increase their aggressiveness [87].

The first clinical trials on the use of ADSCs in anti-cancer therapy have already begun and are described in more detail in Table 1.

The anti-cancer effects of ADSCs include actions on cancer cells and the tumor microenvironment involving the ECM, blood vessels, fibroblasts, endothelial cells, immune system cells, and other cells. Most genes that play a role in communication between ADSCs and fibroblasts or endothelial cells are related to the ECM and vascularization (genes encoding TIMPs; metalloproteinases (MMPs); collagens; members of a disintegrin and metalloproteinase with thrombospondin motifs (ADAMTS), VEGF, and fibroblast growth factor (FGF) families [89]). Intracellular adhesion molecule 1 (ICAM-1), a cytokine produced by ADSCs, increases the expression of the RANTES factor (also known as chemokine ligand 5, CCL5), which in turn increases the expression of metalloproteinases MMP2 and MMP9. These enzymes, through the proper degradation of the ECM, allow cells to migrate and grow and therefore facilitate healing processes [68]. ADSCs also have the ability to stimulate other human cells to express genes for MMPs. In a study by Tilotta et al., the ADSC secretome increased the gene expression of *MMP13* and *ADAMTS5* in human nucleus pulposus cells after IL-1β stimulation [90].

The effect of ADSCs on matrix organization may be of particular interest in cancers where ADSCs are a natural component of the tumor environment, such as breast [91,92], pancreatic [71], or colorectal cancers [93]. The ECM, as an environment for the cells suspended in it, also plays an important role in tissue regeneration processes, including effective wound healing [94]. The tumor ECM may become a target for future anti-cancer therapies [95], potentially also in hepatocellular carcinoma [96].

In contrast to these data, there are some doubts arising from observations that the ADSC secretome may both inhibit and support cancer cell growth (Table 2).

The potential stimulatory effects of ADSCs on cancer cell growth have been demonstrated in several studies [98,99,104,106], especially in ovarian [103,107,108,109] and breast cancers [100,101,102,110,111,112], with some suggestions that this effect of ADSCs may result from their influence on the microenvironment [113]. Moreover, ADSCs co-cultured with melanoma cells from different lines (G-361, SK-Mel-5, MeWo, and A2058) increased the migration capacity of malignant cells, which was correlated with the overexpression of genes such as *CXCL12*, *PTGS2*, *IL-6*, *IL-8*, and, most notably, *HGF*, and increased the expression of CCL2, IL-6, IL-8, VEGF, MMP-2, and the EMM-PRIN proteins [66]. Visceral ADSCs obtained from colorectal cancer patients promoted cancer cell proliferation in in vitro and in vivo xenograft models [93]. Hypoxia-induced ADSCs produced interleukin 10 (IL-10), which promoted the growth of Burkitt’s lymphoma cells [114].

The suggestions of differences in the metabolic profile and interactions with the microenvironment of certain ADSC subpopulations may shed light on the problem of ADSC interactions with cancer cells, especially breast cancer, as adipose tissue grafts are used in breast reconstruction after mastectomy [115,116]. Furthermore, ADSCs derived from mammary tissue may constitute a subpopulation of cells underrepresented in breast cancer studies [117], and this population may have pro-cancerous properties [72,117]. It has been found that mammary tissue ADSCs from patients with breast cancer derived from locations close to and distant from the tumor site show disparities in RNA expression and differentiation potential [72]. 

**Figure 1:**
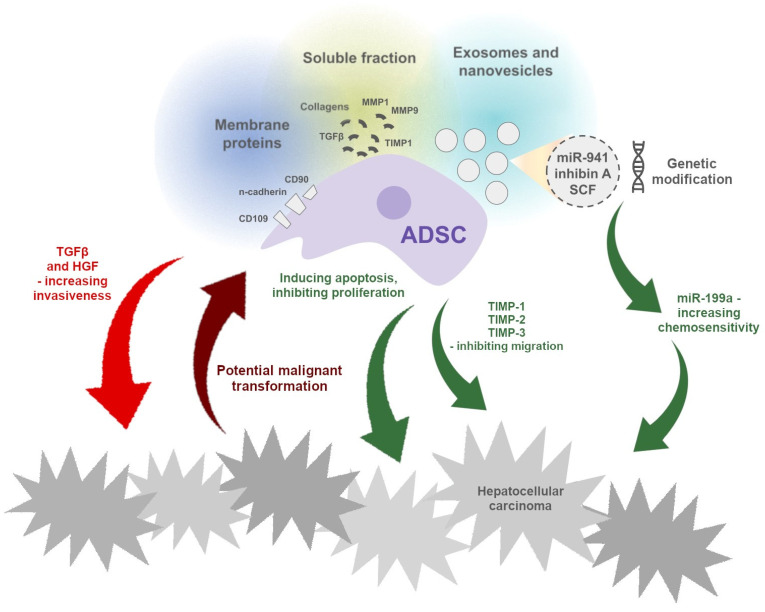
Indicators of the potential mechanisms of the pro- and anti-cancer effects of ADSCs, as well as the possible impact of cancer cells on ADSCs, promoting their neoplastic transformation. Unspecified factors secreted by ADSCs cause apoptosis and the inhibition of cancer cell proliferation in co-cultures in vitro. On the other hand, cancer cells can, through an unknown mechanism, cause the malignant transformation of ADSCs, manifested by the expression of a cancerous phenotype.

To summarize, the effect of ADSCs, a possible anti-cancer agent, on the growth of malignant tumors is not entirely clear, and studies have yielded conflicting results (Figure 1). It is likely that the outcome depends on the patient and the type of tumor.

### 4.1. The Effect of ADSCs on Hepatocellular Carcinoma Cells

Some preclinical studies have suggested the potential use of human ADSCs or ADSC-derived conditioned media in the treatment of hepatocellular carcinoma due to their anti-proliferative effects on multiple hepatocellular carcinoma cell lines, such as HepG2 [83,84], Huh7, SMMC7721, Bel7402 [83], and PLC-PRF-5 cells [84]. ADSCs also inhibited HCC cell migration in HepG2 and PLC-PRF-5 cell cultures. Migration inhibition may have been related to the overexpression of a tissue inhibitor of metalloproteinases [84]. Human ADSCs pretreated with curcumin reduced the proliferation, migration, and invasion of HepG2 hepatocellular carcinoma cells and induced their apoptosis [85]. A similar effect was described for rat ADSC-derived conditioned media and the HepG2 cell line [118] or human ADSC-derived conditioned media and the SMMC7721 cell line (via Akt signaling) [83]. Contrary to these findings, a conditioned medium from canine ADSCs enhanced the proliferation and invasion of the canine HCC cell line [119]. The effects of ADSCs on hepatocellular carcinoma cells are summarized in Table 3.

On the other hand, several studies have described a potential reverse effect of hepatocellular carcinoma cells on ADSCs, namely the risk of malignant transformation of stem cells. The co-culturing of the Huh-7 hepatocellular carcinoma cell line and ADSCs resulted in the acquisition of carcinogenic features in ADSCs, such as higher gene expression of pluripotency markers (*OCT-4* and *NANOG*), proliferation markers (*KRAS* and *CDK6*), and adhesion markers (*EPCAM* and *CD44*), as well as the downregulation of differentiation markers [120]. Moreover, co-culture with Huh-7 improved the proliferation and migration capacity of ADSCs and induced the expression of miRNA related to the oncogenic miR-17-5p and the suppressor miR-615-5p moieties [120]. In addition, Huh-7.5 hepatocellular carcinoma cells co-cultured with ADSCs supported their differentiation toward the hepatic cell line [121].

### 4.2. ADSCs as a Potential Complementary Treatment in Standard Therapies

**Figure 2:**
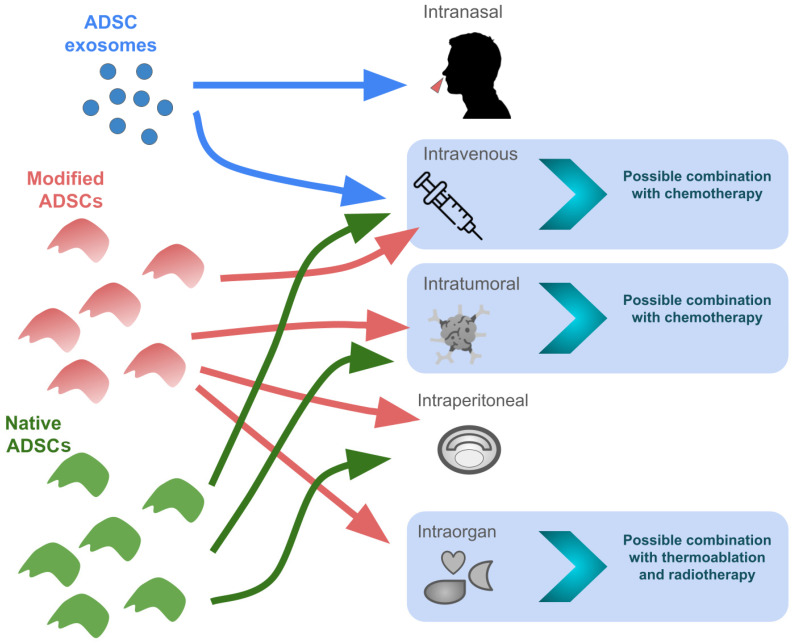
Potential ways of administrating native and modified ADSCs and ADSC exosomes independently or in combination therapies. The intranasal administration of ADSC-derived exosomes has been proven to be effective in the treatment of ischemic nerve damage in mice [125], but their effectiveness in the treatment of cancer has not yet been confirmed.

The ability of ADSCs to migrate towards cancer cells has created the opportunity to use ADSCs and ADSC-derived extracellular vesicles as drug carriers (Figure 2). Some studies have confirmed this concept in vitro and in vivo [122,123]. Human ADSCs transfected with the TRAIL migrated toward hepatocellular carcinoma cells, induced apoptosis, and inhibited the proliferation of HCC cells in vitro, as well as reduced the tumor weight and metastatic abilities in an in vivo mouse model [124].

It has been shown that ADSCs and their extracellular vesicles tend to accumulate in damaged livers. Human ADSC-derived nanovesicles tended to accumulate more often in fibrotic livers compared to normal livers [50]. This effect might be due to the increased activity of hepatic macrophages in damaged livers [50]. Murine ADSC-derived extracellular vesicles were able to incorporate into cells and reduced HepG2 cell growth [126]. In the same study, extracellular vesicles derived from ADSCs were able to encapsulate astaxanthin, suggesting their possible use as drug carriers [126], which, in addition to the ability to migrate toward a damaged liver, may improve the drug distribution profile. A similar effect was described in breast cancer cells. ADSCs were able to carry and release paclitaxel, a standard chemotherapy drug in breast cancer, thus inhibiting breast cancer cell proliferation in vitro, whereas in the case of in vivo xenografts, paclitaxel-loaded ADSCs inhibited tumor growth [127].

ADSCs may also increase the sensitivity to commonly used HCC drugs. Human ADSCs transfected with miRNA plasmids were able to deliver miRNA into hepatocellular carcinoma cells, which increased their sensitivity to 5-fluorouracil (a chemotherapy agent) and sorafenib [128], a drug classically used in HCC, in vitro. Moreover, these plasmids, when used with sorafenib, demonstrated anti-cancer effects in a mouse model of hepatocellular carcinoma [128]. A similar effect of ADSC exosomes was proven for doxorubicin, where exosomes derived from ADSCs transfected with miR-199a increased the sensitivity of PLC/PRF/5 hepatocellular carcinoma cells for chemotherapy, which resulted in the inhibition of proliferation and the induction of apoptosis [129]. In a study on anti-tumor chemotherapy with doxorubicin, ADSCs were shown to display a similar half-maximal inhibitory concentration (IC50) for doxorubicin between serial passages, suggesting their potential role in further in vitro studies on anti-cancer drugs as a stable control [130].

ADSCs could improve other types of HCC therapies, such as thermal ablation or radiotherapy. Porcine ADSCs transfected with iron oxide-coated gold nanoparticles were shown to accumulate in damaged mouse livers and were able to mediate laser irradiation, resulting in the thermal ablation of HepG2 hepatocellular carcinoma cells in vitro [131]. When combined with radiotherapy, human ADSCs enhanced its anti-cancer effect. The results showed reduced viability, proliferation, sphere formation, and colony formation in liver cancer cells in vitro, as well as impaired migration, wound healing, and invasion of hepatocellular carcinoma cells [86]. The effect was consistent with in vivo experiments in a mouse model, where the combination of radiotherapy and ADSCs resulted in reduced tumor volume and weight, lower proliferation index, and a higher percentage of apoptotic cancer cells [86].

## 5. Conclusions

ADSCs have a wide variety of potential applications, including drug delivery, chemosensitization, liver regeneration after surgery, thermal ablation, and radiotherapy. However, most studies have referred to in vitro interactions between ADSCs and human cancer cells or in vivo animal models. The studies have usually focused on cell-to-cell interactions between ADSCs and certain human HCC cell lines. The potential of such an HCC model is limited since a cell line is usually derived from a single patient and is homogeneous and therefore does not represent cancer heterogeneity. Moreover, recent studies have suggested that one of the most commonly used cell lines, HepG2, may not represent hepatocellular carcinoma but another liver malignancy: hepatoblastoma [132]. Most studies on the effects of ADSCs in cancer models do not consider immunomodulation and interactions with the extracellular matrix of the tumor, which may be important complementary effects to the usually reported cell-to-cell interactions between ADSCs and cancer cells. There are no clinical studies testing the use of ADSCs in hepatocellular carcinoma or describing their role in other cancers. In conclusion, comprehensive studies, including in vitro and in vivo experimental models, as well as clinical trials, are essential to prove the concept of the therapeutic potential of ADSCs in HCC in the near future. However, regardless of the uncertainties described here, the use of stem cells in hepatocellular carcinoma may still become a promising addition to standard therapies due to their regenerative potential, positive effect on extracellular matrix remodeling, and anti-apoptotic effect on HCC cells.

## Figures and Tables

**Table 1:** The use of ADSCs in clinical trials for various types of cancer, accessed at clinicaltrials.gov on 24 April 2024.

Study Number	Cancer Type	ADSC Source	ADSC Quantity	Administration	Results	Ref.
NCT 05789394	Glioblastoma multiforme	Allogeneic transplant	5 × 10^6^ −2 × 10^7^	Intratumoral	Ongoing study	[88]
NCT 04087889	Pancreatic cancer	Allogeneic transplant from a first-degree relative	2 × 10^8^	Intravenous	Ongoing study	Unpublished
NCT 02068794	Ovarian cancer	Not specified	Not specified	Intraperitoneal	Ongoing study	Unpublished

**Table 2:** Selected substances produced by ADSCs identified as having either stimulatory (↑) or suppressive (↓) effects on cancer cell growth.

Factor	Effect on Cancer Cell Growth	Ref.
Il-6	↑	[97,98,99]
Adipsin	↑	[100]
Stem cell factor (SCF)	↑	[101,102]
TGFβ	↑	[103]
Cysteine-rich angiogenic inducer 61 (Cyr61)	↑	[104]
Pro-apoptotic factors (not specified)	↓	[76,77,81,82]
miR-941	↓	[105]

**Table 3:** Summary of the experimental studies related to the harmful and beneficial effects of ADSCs on hepatocellular carcinoma cell lines.

Effect	Method	Cell Line	Ref.
Inhibition of proliferation	Co-culture with ADSCs	HepG2	[84,85]
		PLC-PRF-5	[84]
	Culture with ADSC-derived conditioned medium	HepG2	[83,84,118]
		Huh7	[83]
		SMMC7721	[83]
		Bel7402	[83]
Induction of apoptosis	Co-culture with ADSCs	HepG2	[84,85]
		PLC-PRF-5	[84]
	Culture with ADSC-derived	SMMC7721	[83]
	conditioned medium	HepG2	[84,118]
		PLC-PRF-5	[84]
Inhibition of migration	Co-culture with ADSCs	HepG2	[84,85]
		PLC-PRF-5	[84]
	Culture with ADSC-derived conditioned medium	HepG2	[84,118]
Reduction of invasiveness	Co-culture with ADSCs	HepG2	[84]
	Culture with ADSC-derived conditioned medium	HepG2	[84,85,118]
Promotion of proliferation and migration	Culture with ADSC-derived conditioned medium	AZACH	[119]

## Data Availability

The data presented in this study are available in the article and will be made available upon reasonable request.
